# Ensemble learning to enhance accurate identification of patients with glaucoma using electronic health records

**DOI:** 10.1093/jamiaopen/ooaf080

**Published:** 2025-08-10

**Authors:** Tushar Mungle, Behzad Naderalvojoud, Chris A Andrews, Hong Su An, Amanda Bicket, Amy Zhang, Julie Rosenthal, Wen-Shin Lee, Chase A Ludwig, Bethlehem Mekonnen, Suzann Pershing, Joshua D Stein, Tina Hernandez-Boussard, SOURCE Consortium, Suzann Pershing, Sophia Y Wang, Sejal Amin, Paul A Edwards, Divya Srikumaran, Fasika Woreta, Jeffrey S Schultz, Anurag Shrivastava, Baseer Ahmad, Louis P Pasquale, Paul Bryar, Dustin French, Rajeev Ramachandran, Brian L Vanderbeek, Preethi Ganapathy, Michael Deiner, Catherine Sun, Jenna Patnaik, Prem Subramanian, Saleha Munir, Wuqaas Munir, Joshua D Stein, Lindsey De Lott, Robert Feldman, Brian C Stagg, Barbara Wirostko, Brian McMillian, Arsham Sheybani, Ji Liu, Soshian Sarrapour

**Affiliations:** Department of Medicine, Stanford University, Stanford, CA 94305, United States; Department of Medicine, Stanford University, Stanford, CA 94305, United States; Department of Ophthalmology and Visual Sciences, University of Michigan, Ann Arbor, MI 48105, United States; Department of Ophthalmology and Visual Sciences, University of Michigan, Ann Arbor, MI 48105, United States; Department of Ophthalmology and Visual Sciences, University of Michigan, Ann Arbor, MI 48105, United States; Department of Ophthalmology and Visual Sciences, University of Michigan, Ann Arbor, MI 48105, United States; Department of Ophthalmology and Visual Sciences, University of Michigan, Ann Arbor, MI 48105, United States; Department of Ophthalmology, Byers Eye Institute, Stanford University, Stanford, CA 94305, United States; Department of Ophthalmology, Byers Eye Institute, Stanford University, Stanford, CA 94305, United States; Department of Ophthalmology, Byers Eye Institute, Stanford University, Stanford, CA 94305, United States; Department of Ophthalmology, Byers Eye Institute, Stanford University, Stanford, CA 94305, United States; Department of Ophthalmology and Visual Sciences, University of Michigan, Ann Arbor, MI 48105, United States; Department of Health Management and Policy, School of Public Health, University of Michigan, Ann Arbor, MI 48109, United States; Department of Medicine, Stanford University, Stanford, CA 94305, United States

**Keywords:** real-world data, ensemble learning, clinical notes, class imbalance, generalizability and fairness

## Abstract

**Objectives:**

Existing ophthalmology studies for clinical phenotypes identification in real-world datasets (RWD) rely exclusively on structured data elements (SDE). We evaluated the performance, generalizability, and fairness of multimodal ensemble models that integrate real-world SDE and free-text data compared to SDE-only models to identify patients with glaucoma.

**Materials and Methods:**

This is a retrospective cross-sectional study involving 2 health systems- University of Michigan (UoM) and Stanford University (SU). It involves 1728 patients visiting eye clinics during 2012-2021. Free-text embeddings extracted using BioClinicalBERT were combined with SDE. EditedNearestNeighbor (ENN) undersampling and Borderline-Synthetic Minority Over-sampling Technique (bSMOTE) addressed class imbalance. Lasso Regression (LR), Random Forest (RF), Support Vector Classifier (SVC) models were trained on UoM imbalanced (imb) and resampled data along with bagging ensemble method. Models were externally validated with SU data. Fairness was assessed using equalized odds difference (EOD) and Target Probability Difference (TPD).

**Results:**

Among 900 and 828 patients from UoM and SU, 10% and 23% respectively had glaucoma as confirmed by ophthalmologists. At UoM, multimodal LR_imb_ (F1 = 76.60 [61.90-88.89]; AUROC = 95.41 [87.01-99.63]) outperformed unimodal RF_imb_ (F1 = 69.77 [52.94-83.64]; AUROC = 97.72 [95.95-99.18]) and ICD-coding method (F1 = 53.01 [39.51-65.43]; AUROC = 90.10 [84.59-93.93]). Bagging (BM = LR_ENN_ + LR_bSMOTE_) improved performance achieving an F1 of 83.02 [70.59-92.86] and AUROC of 97.59 [92.98-99.88]. During external validation BM achieved the highest F1 (68.47 [62.61-73.75]), outperforming unimodal (F1 = 51.26 [43.80-58.13]) and multimodal LR_imb_ (F1 = 62.46 [55.95-68.24]). BM EOD revealed lower disparities for sex (<0.1), race (<0.5) and ethnicity (<0.5), and had least uncertainty using TDP analysis as compared to traditional models.

**Discussion:**

Multimodal ensemble models integrating structured and unstructured EHR data outperformed traditional SDE models achieving fair predictions across demographic sub-groups. Among ensemble methods, bagging demonstrated better generalizability than stacking, particularly when training data is limited.

**Conclusion:**

This approach can enhance phenotype discovery to enable future research studies using RWD, leading to better patient management and clinical outcomes.

## Introduction

Machine learning (ML) is increasingly used in the healthcare domain to identify patients of interests. In the field of ophthalmology, these ML models often rely upon electronic health record (EHR) data, and many only consider structured data, such as: International Classification of Diseases (ICD) or Current Procedural Terminology (CPT) codes, demographic information, lab test results, and vital signs.^1^^,^^2^ However, these unimodal-data models may not be capable of providing a complete patient view and the care they are receiving, and this may limit their clinical utility. Furthermore, phenotypes or condition of interest that are not overly prevalent^3^ may suffer from class imbalance issues making identification of patients with these less common but serious condition more difficult.^4^^,^^5^ This imbalance affects precision and recall resulting in costly misclassifications. While instance weighting and sampling techniques help mitigate these concerns, clearly additional approaches are needed to enhance model performance to an accuracy level for phenotyping task.^5^

Efforts to improve the predictive capabilities of ML models for decision-making can be achieved by tapping into additional EHR data.^5^^,^^6^ Clinical information not captured in structured data is typically found in the free text of patient’s clinical notes.^7–9^ Synthesizing such complementary information and incorporating them into ML models have provided better results for generating real-world evidence.^8^ One way to utilize both structured and unstructured data during ML model development is data fusion techniques. These techniques combine different data modalities to generate unified feature space or data representation for single model training to improve performance.^10^^,^^11^

Another effective solution for enhancing model performance is ensemble learning (EL) techniques such as bagging and stacking.^12–14^ These approaches involve combining multiple weak classifiers, each trained on different subsets of features or samples. EL with strong classifiers trained on different feature sets improves performance in clinical applications.^13^ However, EL alone does not address the class imbalance problem, with skewness still favoring the majority class. Additionally, assessing generalizability and fairness of models deploying resampling and EL methods to address class imbalance in the clinical domain are yet unexplored.

In this study, we aim to develop and evaluate a comprehensive approach to fairly and accurately classify patients with and without glaucoma, a sight threatening ocular disease that affects more than 3 million Americans,^15^ using multimodal real-world data across diverse populations. We investigated strategies to address class imbalance and improve algorithmic fairness. To our knowledge, this is the first study to systematically evaluate the performance and fairness of multimodal ensemble learning models in this context. We hypothesize that combining machine learning with data resampling within a multimodal data setting can significantly improve model performance, generalizability, and fairness which can help clinicians to leverage real-word data to answer clinical questions.

## Materials and methods

### Data source

This study includes EHR data from individuals receiving eye care at two tertiary care health systems participating in the Sight Outcomes Research Collaborative (SOURCE): the University of Michigan (UoM, data from 2012-2021) and Stanford University (SU, data from 2013-2021). SOURCE has deidentified longitudinal data on eye care recipients from 20 health systems throughout the United States on EPIC-EHR.^16^ Data are deidentified using Datavant software.^17^ Several studies have utilized SOURCE data to study patients with ocular diseases.^18–20^ The Institutional Review Boards at UoM and SU approved this study.

The study included patients aged 40-80 years old with at least 2 visits to eye care professionals. The data variables included ICD billing codes, patient demographics, eye exam findings, outpatient medications and surgical treatments from various EHR locations as described by Stein et al.^21^ We augmented the structured data with free text data from outpatient clinic visit notes. Eye exam findings and treatment data were aggregated into patient-level features. The final dataset consists of 82 structured-data features and the free text of the most recent eye visit note for each patient. The study included data from 1728 randomly selected adult patients, of which 900 were from UoM and 828 from SU.

Two board certified ophthalmologists at UoM and SU reviewed the EHRs and classified each patient as having “definite,” “possible,” or “no evidence” of glaucoma. A third board certified ophthalmologist, at each site, adjudicated any disagreements. “Possible glaucoma” captured the cases that ophthalmologist could not decide if the patient did or did not have glaucoma based on the clinical data. This is different from “glaucoma suspect” who were patients with risk factors for glaucoma but no evidence of structural or functional damage present. These patients were grouped with patients with no glaucoma. Patients were classified as having glaucoma if at least one eye showed findings consistent with glaucoma (**Figure S1**). The UoM dataset was divided into training (70%) and testing (30%) sets using simple random sampling.

### Clinical note embeddings

The free text of clinical notes were processed using the BioClinicalBERT model,^22^ to extract embeddings. BioClinicalBERT is a transformer model fine-tuned on EHR data. Token sequences with a maximum length of 512 were generated for each clinical note to produce embeddings from the model. Embeddings were extracted from the last hidden layer, resulting in vectors of dimension 1x768. For notes exceeding the length limit (**Figures S2 and S3**), multiple embedding layers (nx768) were generated and subsequently averaged to produce a final feature vector of 1x768 for each patient as described in Fanconi et al.^23^

### Model development for imbalanced multimodal data

Early data fusion was used to combine clinical note embeddings and structured data fields before training the models. It ensured that both types of data modalities contributed to the model’s learning process from the outset. To mitigate the limitations of an imbalanced dataset, under-sampling and over-sampling techniques were tested. Under-sampling methods like NearMiss, EditedNearestNeighbors (ENN), and Instance Hardness Threshold reduce majority class instances, while over-sampling techniques such as Synthetic-Minority Oversampling Technique (SMOTE), Adaptive Synthetic (ADASYN), and Borderline SMOTE (bSMOTE) increase minority class instances.^24^

Using imbalanced, undersampled, and oversampled data, ML models were developed. These included Lasso Regression (LR) for feature selection and regularization, Random Forest (RF) for its ensemble capabilities, and Support Vector Classifier (SVC) for optimizing classification hyperplanes. These models were trained using default hyperparameters. Two ensemble learning techniques were applied to enhance accuracy. Bagging involved training multiple models on different resampled data and aggregating their predicted probabilities, thereby reducing variance and overfitting.^25^ Stacking combined predictions from diverse base models through a meta-model, optimizing the combination of individual model strengths and capturing more complex relationships, to enhance predictive performance.^25^

### Experimental setup

The base models (LR, RF, and SVC) using imbalanced unimodal data (LR_imb_, RF_imb_, SVC_imb_) were developed. Next, the base models were trained using multimodal (structured and unstructured) data in combination with resampling techniques. The above-mentioned under- and over-sampling techniques were used on the training data to identify the best performing resampling technique for model training. Finally, ensemble methods where base models trained with the original imbalanced datasets and those trained with resampling techniques were combined using bagging and stacking approaches.

### Evaluation metrics

Model performance was evaluated using precision, recall, and F1 score all calculated using a risk threshold of 0.5, and the area under the receiver-operating curve (AUROC).^26^ To generate 95% confidence intervals, bootstrapping was conducted by independently resampling 2000 times with replacement from the original test dataset. Non-parametric tests (Wilcoxon and Kruskal Wallis) were used to compare values between two to more groups. We used UoM test set for evaluation that was masked from the UoM training set.

### Feature importance analysis

To evaluate the importance of various features for the models developed, we employed two distinct approaches. First, we analyzed the top 10 coefficients from our best-performing model trained on the UoM data. This method allowed us to interpret the influence of each feature on the model’s predictions, quantifying the strength of their relationships with the outcome variable. Second, we utilized SHapley Additive exPlanations (SHAP) to further investigate feature significance across the UoM test data.^27^ SHAP values provide a comprehensive understanding of how each feature impacts model classification, with larger absolute values indicating greater influence. Positive SHAP values (greater than 0) associated with high covariate values (red color) and negative SHAP values (less than 0) linked to low covariate values (blue color) were interpreted as features that contribute to the likelihood of a patient having glaucoma.

### Generalizability, fairness, and accuracy evaluation

To evaluate the generalizability of the models trained on data from UoM, we performed an external validation using data from SU. The best-performing model, identified based on both internal and external validation, was further examined to check for potential biases. Same model was considered to evaluate fairness using Equalized Odds Difference (EOD) across different demographic groups (sex, race, and ethnicity). EOD measures the difference in error rates (such as True Positive Rate and False Positive Rate) between demographic groups.^26^^,^^28^ A lower EOD value indicates that model performs more equitably across patients of different demographic profiles.

To complement the fairness evaluation with a focus on probabilities, we introduce a concept of Target Probability Difference (TPD). TPD is defined as the absolute difference between true labels and predicted probabilities, evaluating the closeness of predicted probabilities to actual outcomes. The key idea is based on foundational work described by Vapnik^29^ which minimizes the difference between target and predicted probabilities during the training phase of machine learning models. Unlike binary metrics that categorize predictions into right or wrong, TPD provides prediction confidence and error margins, which cannot be achieved using standard fairness metrics, such as EOD. A lower TPD value signifies a model’s better capability to make predictions across different demographic sub-groups.

**Figure 1.**
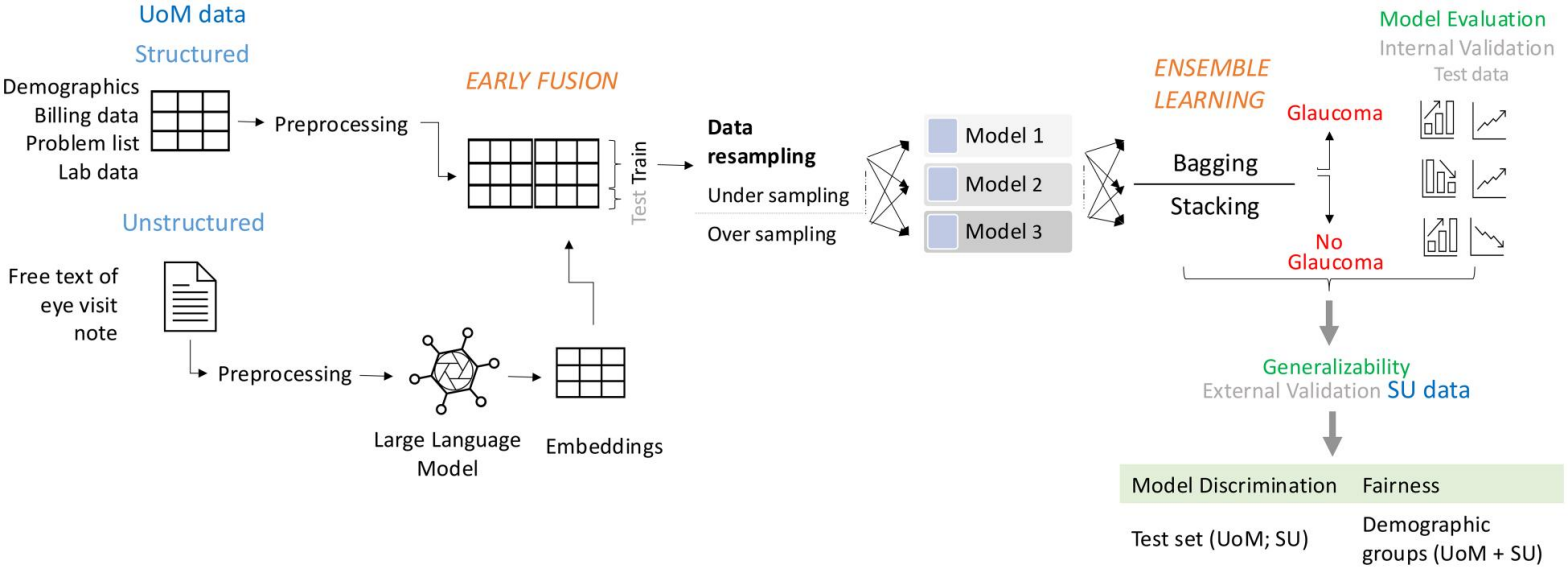
Overall methodology for the study..

Study methodology is described in Figure 1.

## Results

ML model development and internal validation was carried out using UoM data of 900 patients, of which 10% (91/900) individuals were identified by the gold standard grading of the ophthalmologists to have glaucoma (**Table S1**). Model performance for unimodal and multimodal data, along with various resampling techniques, are detailed in Table 1.

**Table 1.** Performance of ICD codes, machine learning and ensemble models trained on University of Michigan dataset incorporating different sampling techniques.^a^

	Model Performance			
	Precision	Recall	F1 score	AUROC
**ICD Codes**	36.67 [25.36-49.31]	95.65 [85.71-100.00]	53.01 [39.51-65.43]	90.10 [84.59-93.93]
	**Uni-modal**			
**Imbalanced Data (imb)**				
LR_imb_	66.67 [47.83-85.71]	69.57 [50.00-87.50]	68.09 [51.28-82.14]	93.86 [84.33-98.90]
RF_imb_	75.00 [55.56-92.87]	65.22 [45.44-84.21]	69.77 [52.94-83.64]	97.72 [95.95-99.18]
SVC_imb_	81.25 [61.11-100.00]	56.52 [36.36-76.47]	66.67 [47.06-81.82]	95.63 [92.05-98.46]
	**Multi-modal**			
**Imbalanced Data (imb)**				
LR_imb_	75.00 [57.13-91.67]	78.26 [61.29-94.12]	76.60 [61.90-88.89]	95.41 [87.01-99.63]
RF_imb_	100.00 [100.00-100.00]	17.39 [4.00-33.33]	29.63 [7.69-50.00]	96.53 [93.47-98.94]
SVC_imb_	72.73 [41.67-100.00]	34.78 [15.79-55.00]	47.06 [24.00-66.67]	96.74 [94.54-98.50]
**EditedNearestNeighbours (ENN)**				
LR_ENN_	61.76 [45.71-78.12]	91.30 [77.78-100.00]	73.68 [59.99-85.29]	97.75 [94.02-99.81]
RF_ENN_	85.71 [64.29-100.00]	52.17 [31.03-72.74]	64.86 [43.46-81.09]	96.87 [94.07-99.10]
SVC_ENN_	63.64 [42.86-83.33]	60.87 [38.89-80.00]	62.22 [43.24-76.60]	96.34 [93.68-98.38]
**Boderline SMOTE (bSMOTE)**				
LR_bSMOTE_	64.52 [48.27-81.26]	86.96 [71.43-100.00]	74.07 [60.00-85.71]	95.30 [87.10-99.54]
RF_bSMOTE_	92.31 [75.00-100.00]	52.17 [31.58-72.73]	66.67 [45.45-82.61]	98.71 [97.40-99.62]
SVC_bSMOTE_	70.59 [47.82-92.31]	52.17 [31.82-71.43]	60.00 [41.02-75.68]	97.22 [95.12-98.76]
**Ensemble Learning**				
**Bagging**				
LR_imb_ + LR_ENN_	72.41 [55.55-88.00]	91.30 [78.56-100.00]	80.77 [67.80-91.23]	97.39 [92.85-99.82]
LR_imb_ + LR_bSMOTE_	69.23 [52.17-86.67]	78.26 [61.29-94.12]	73.47 [58.54-85.71]	95.42 [86.88-99.66]
LR_ENN_ + LR_bSMOTE_	73.33 [57.58-88.46]	95.65 [85.71-100.00]	83.02 [70.59-92.86]	97.59 [92.98-99.88]
**Stacking**				
LR_imb_ + LR_ENN_	84.62 [62.50-100.00]	100.00 [100.00-100.00]	91.67 [76.92-100.00]	99.78 [99.06-100.00]
LR_imb_ + LR_bSMOTE_	81.82 [54.55-100.00]	81.82 [57.14-100.00]	81.82 [57.14-96.01]	99.34 [97.95-100.00]
LR_ENN_ + LR_bSMOTE_	91.67 [72.73-100.00]	100.00 [100.00-100.00]	95.65 [84.21-100.00]	99.78 [99.06-100.00]

a LR: Lasso Regression; RF: Random Forest; SVC: Support Vector Classifier; AUROC: Area Under the Receiver-Operating Curve; SMOTE: Synthetic-Minority Oversampling Technique. Threshold value of 0.5 was used to evaluate the metrices. Numbers in the bracket represent 95% confidence interval.

### Internal validation using data from UoM

The traditional ICD-code based phenotyping (rule-based) has a very low F1 score of 53.01 [39.51-65.43] due to low precision (36.67 [25.63-49.31]). For unimodal data from the internal (UoM) test set, the RF outperformed LR and SVC with an F1 score of 69.77 [52.94-83.64] and an AUROC of 97.72 [95.95-99.18] (Table 1). Using multimodal data, the LR demonstrated considerable improvement over the unimodal LR, achieving an F1 score of 76.60 [61.90-88.89] and an AUROC of 95.41 [87.01-99.63].

To address class imbalance, we evaluated various resampling techniques on training data with a separate LR model. ENN and bSMOTE outperformed the other respective resampling techniques achieving higher F1 scores (**Table S2**) and were selected for further experiments. Incorporating ENN—(LR_ENN_, RF_ENN_, SVC_ENN_) and bSMOTE—(LR_bSMOTE_, RF_bSMOTE_, SVC_bSMOTE_) maintained overall model performance same. LR_ENN_ and LR_bSMOTE_ achieved an F1 scores of 73.68 [59.99-85.29] and 74.07 [60.00-85.71], respectively (Table 1).

Ensembles of LRs outperformed RF and SVC using different sampling techniques and was considered for further experiments. Bagging LR_ENN_ + LR_bSMOTE_, substantially improved the F1 score to 83.02 [70.59-92.86] and achieved an AUROC of 97.59 [92.98-99.88]. Stacking ensemble approach with an additional LR as the meta-classifier, the LR_ENN_ + LR_bSMOTE_ achieved the highest F1 score of 95.65 [84.21-100.00] and an AUROC of 99.78 [99.06-100.00].

### Model interpretability and explainability

**Figure 2.**
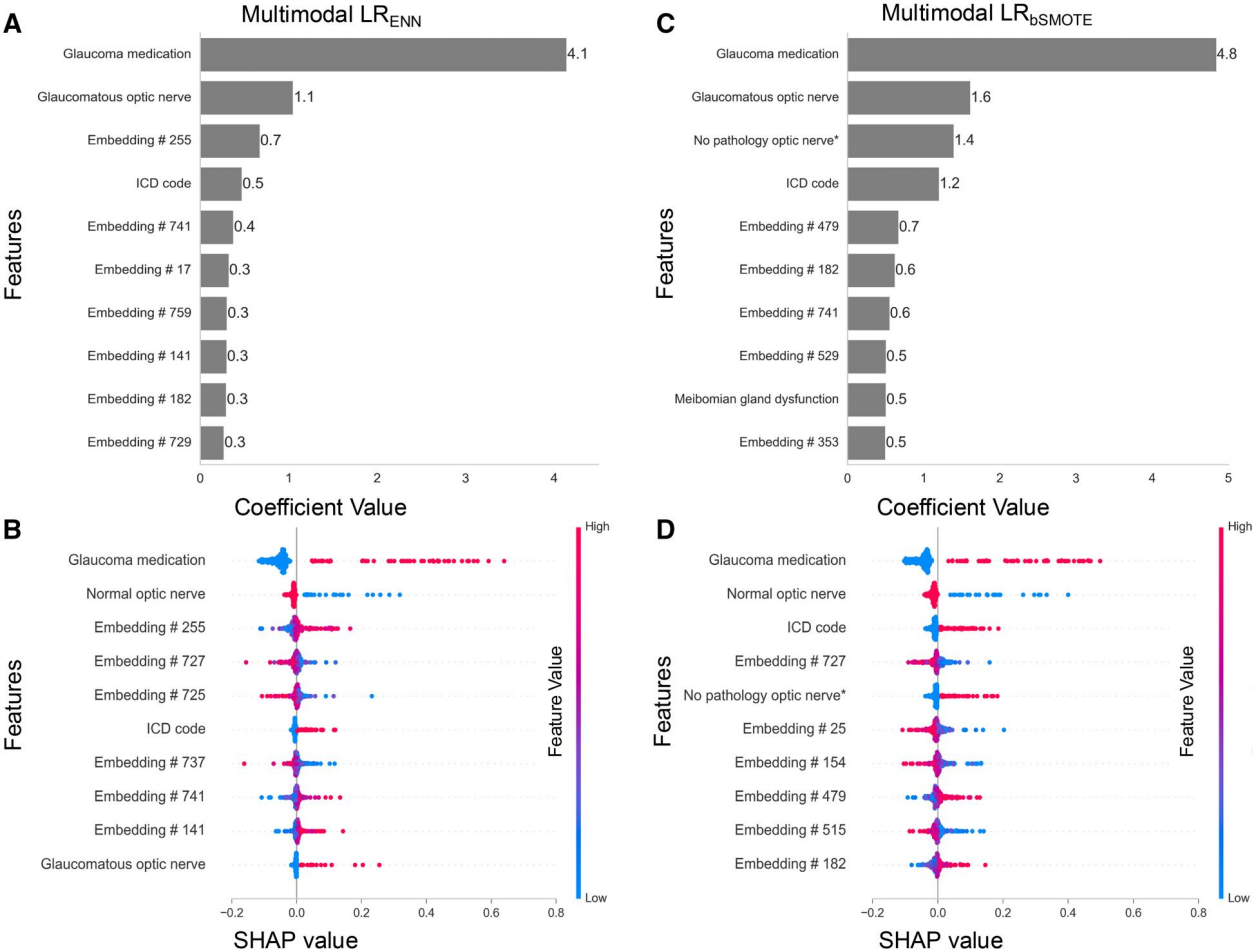
Model interpretability and explainability illustrating the contribution of various features in distinguishing patients with and without glaucoma for multimodal LR models trained with different sampling techniques on internal data (University of Michigan). (A) and (B) represents model coefficients and SHAP for LR_ENN_ respectively; (C) and (D) represents model coeffieicients and SHAP values for LR_bSMOTE_ respectively. LR: Lasso Regression; ENN: EditedNearestNeighbours; bSMOTE: Borderline Synthetic-Minority Oversampling Technique; SHAP: SHapley Additive exPlanations*;* *“No pathology, optic nerve” captures information such as “no disc hemorrhage.”

The performance of bagging and stacking techniques was highest for the combination of LR_ENN_ and LR_bSMOTE_ models (Table 1) and were considered for interpretability and explainability analysis. As illustrated in Figure 2, both models exhibit distinct feature importance and SHAP values, highlighting their unique contributions to the classification process. For the LR_ENN_ model (Figure 2A), the top features include glaucoma medication (coefficient value: 4.1), glaucomatous optic nerve (coefficient value: 1.1), and ICD code (coefficient value: 0.5). Other notable features include various clinical note embeddings. Additionally, their respective SHAP values indicates their importance in the model’s performance (Figure 2B). In contrast, the LR_bSMOTE_ model (Figure 2C) emphasizes different features, with glaucoma medication (coefficient value: 4.8) again being the most significant, followed by glaucomatous optic nerve (coefficient value: 1.6) and no pathology optic nerve (coefficient value: 1.4). The ICD code and meibomian gland dysfunction also plays a crucial role along with other embeddings that contribute to the model’s performance. Similarly, SHAP values shows different feature contributions for LR_bSMOTE_ as compared to LR_ENN_ (Figure 2D). Although some features are common among the both the models, their coefficient and SHAP values are different.

### External validation using data from SU

The UoM trained models were externally validated on the SU dataset (828 patients with 23% of whom were identified to have glaucoma) (**Table S1**). Once again, the multimodal data trained LR demonstrated a higher F1 score as compared to the unimodal data trained models, 62.46 [55.95-68.24] vs 51.26 [43.80-58.13], and outperformed RF and SVC (Table 2). Models trained with resampled data, LR_ENN_ and LR_bSMOTE_, showed better F1 scores, 65.42 [59.35-71.13] and 68.35 [62.57-73.60], respectively, as compared to LR_imb_. Additionally, the AUROC for LR_imb_, LR_ENN_, LR_bSMOTE_ showed incremental performance improvements. Bagging, LR_ENN_ + LR_bSMOTE_, elevated the F1 score to 68.47 [62.61-73.75] and AUROC to 89.33 [86.36-92.11] values as compared to the other models. However, the stacking ensemble approach did not improve performance as compared to imbalance or Bagging models.

**Table 2.** External Validation of models trained using University of Michigan data on data from Stanford University dataset.^a^

	Model Performance			
	Precision	Recall	F1 score	AUROC
	**Unimodal**			
**Imbalanced Data (imb)**				
LR_imb_	80.68 [72.22-88.76]	37.57 [30.99-44.51]	51.26 [43.80-58.13]	90.85 [88.28-93.12]
RF_imb_	92.45 [84.78-98.36]	25.93 [19.87-32.52]	40.50 [32.48-48.25]	89.84 [86.99-92.47]
SVC_imb_	95.24 [87.23-100.00]	21.16 [15.46-27.17]	34.63 [26.56-42.45]	88.46 [85.52-91.32]
	**Multi-modal**			
**Imbalanced Data**				
LR_imb_	77.34 [69.92-84.75]	52.38 [45.09-59.14]	62.46 [55.95-68.24]	89.10 [86.25-91.74]
RF_imb_	100.00 [0.00-100.00]	1.06 [0.00-2.75]	2.09 [0.00-5.35]	82.31 [79.29-85.27]
SVC_imb_	86.84 [75.00-97.14]	17.46 [12.31-22.80]	29.07 [21.40-36.43]	76.70 [72.36-80.61]
**EditedNearestNeighbours (ENN)**				
LR_ENN_	79.55 [72.60-86.57]	55.56 [48.76-62.50]	65.42 [59.35-71.13]	88.29 [85.06-91.21]
RF_ENN_	89.47 [78.79-97.62]	17.99 [12.50-23.66]	29.96 [21.83-37.71]	83.90 [80.71-86.80]
SVC_ENN_	77.03 [67.24-85.94]	30.16 [23.47-36.42]	43.35 [35.56-50.38]	75.67 [71.26-79.72]
**Boderline SMOTE (bSMOTE)**				
LR_bSMOTE_	72.62 [65.87-79.14]	64.55 [57.62-71.04]	68.35 [62.57-73.60]	88.22 [85.27-90.89]
RF_bSMOTE_	100.00 [100.00-100.00]	9.52 [5.53-13.69]	17.39 [10.47-24.08]	90.29 [87.60-92.71]
SVC_bSMOTE_	81.93 [73.42-89.48]	35.98 [28.99-42.78]	50.00 [42.40-56.93]	76.43 [72.05-80.55]
**Ensemble Learning**				
**Bagging**				
LR_imb_ + LR_ENN_	82.95 [76.36-89.39]	56.61 [49.49-63.13]	67.30 [61.17-72.83]	89.55 [86.64-92.22]
LR_imb_ + LR_bSMOTE_	74.32 [67.15-81.41]	58.20 [50.62-64.90]	65.28 [59.00-70.82]	88.82 [85.99-91.48]
LR_ENN_ + LR_bSMOTE_	79.17 [72.26-85.72]	60.32 [53.23-67.02]	68.47 [62.61-73.75]	89.33 [86.36-92.11]
**Stacking**				
LR_imb_ + LR_ENN_	85.29 [78.12-92.14]	46.03 [39.15-52.94]	59.79 [53.09-65.88]	89.37 [86.39-92.16]
LR_imb_ + LR_bSMOTE_	78.57 [70.75-86.18]	46.56 [39.36-53.48]	58.47 [51.43-64.62]	88.96 [86.10-91.64]
LR_ENN_ + LR_bSMOTE_	84.62 [77.36-91.51]	46.56 [39.64-53.30]	60.07 [53.47-66.23]	89.28 [86.26-92.04]

a LR: Lasso Regression; RF: Random Forest; SVC: Support Vector Classifier; AUROC: Area Under the Receiver-Operating Curve; ENN: Edited Nearest Neighbours; bSMOTE: Borderline Synthetic-Minority Oversampling Technique. Threshold value of 0.5 was used to evaluate the metrices. Numbers in the bracket represent 95% confidence interval.

### Performance evaluation

To investigate decline in model performance for SU data validation (Table 2), the classification probability distribution for the glaucoma and non-glaucoma classes across unimodal and multimodal settings using the UoM and SU datasets (**Figure S4**) was evaluated. The bagging, LR_ENN_ + LR_bSMOTE_, was selected as it performed best across both sets of data. During internal and external validation, the violin plot density peak shifts to the top for glaucoma class, indicating better classification (**Figure S4**). **Figure S5** illustrates the prevalence of glaucoma across all the covariates (except continuous). A distribution disparity is observed between both the datasets highlighting one of the potential reasons for performance difference (**Figure S5**; Kolmogorov-Smirnov test; *P*-value < .0001).

### Model fairness

To provide context to our fairness analysis, the patient population with glaucoma at UoM consisted of 68% White, 21% Black, and 6.6% Asian Americans, with a slight male majority (52%). The patient population with glaucoma at SU had a racial composition of 43% White, less than 3% Black, 34% Asian, and a female majority (59%). Both the sites had a predominantly non-Hispanic patient population. The bagging LR_ENN_ + LR_bSMOTE_ model consistently demonstrated the lowest disparities across demographic variables (Table 3), showing a difference of only 0.07 [0.00-0.22] (UoM) and 0.06 [0.01-0.16] (SU) for sex, and a difference of 0.54 [0.04-1.00] (UoM) and 0.29 [0.07-0.68] (SU) for race. For ethnicity, the model showed biased predictions at UoM (EOD = 0.49 [0.03-1.00]) whereas it was less biased with low disparity at SU (EOD = 0.23 [0.05-0.56]).

**Table 3.** Fairness analysis for ML models using equalized odds difference.^a^

Demographic Variable	Equalized odds Difference	
	UoM	SU
**Unimodal LR_imb_**		
Sex	0.17 [0.02-0.46]	0.06 [0.01-0.15]
Race	0.75 [0.24-1.00]	0.28 [0.06-0.68]
Ethnicity	0.65 [0.17-1.00]	0.23 [0.04-0.54]
**Multimodal LR_imb_**		
Sex	0.15 [0.01-0.42]	0.06 [0.01-0.17]
Race	0.71 [0.13-1.00]	0.30 [0.07-0.63]
Ethnicity	0.63 [0.11-1.00]	0.23 [0.05-0.57]
**Bagging LR_ENN_ + LR_bSMOTE_**		
Sex	0.07 [0.00-0.22]	0.06 [0.01-0.16]
Race	0.54 [0.04-1.00]	0.29 [0.07-0.68]
Ethnicity	0.49 [0.03-1.00]	0.23 [0.05-0.56]

a LR: Lasso Regression; UoM: University of Michigan; SU: Stanford University; imb: Imbalanced data; ENN: EditedNearestNeighbours; bSMOTE: Borderline Synthetic-Minority Oversampling Technique. Values are represented as mean [95% confidence interval] using bootstrapping as described in Method section.

**Figure 3.**
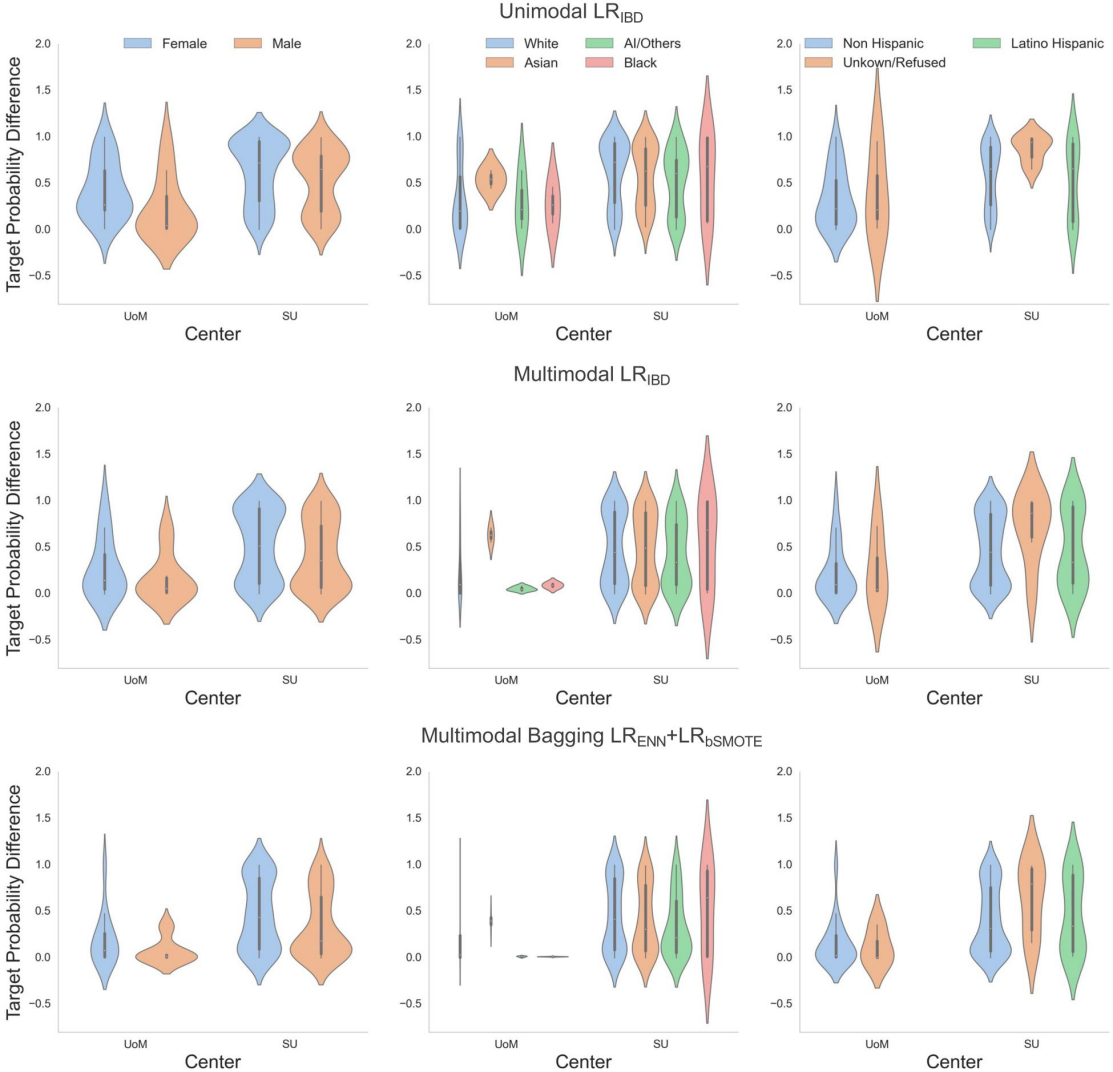
Comparison of fairness for various models using target probability difference for demographic sub-groups. UoM: University of Michigan; SU: Stanford University; LR: Lasso Regression; ENN: EditedNearestNeighbours; bSMOTE: Borderline Synthetic-Minority Oversampling Technique. Note: The extension of the violin plot below 0 or above 1 does not represent negative or greater than 1 probability values but a feature of kernel density estimation used to indicate data distribution.

In addition to the above fairness differences, the accuracy and confidence (fairness sub-analysis) of the model’s probabilities among patients identified with glaucoma with respect to socio-demographic characteristics using TPD is described in Figure 3 (**Table S3**). No disparities were observed in the approach on internal validation data from UoM. On external validation, a significant disparity by sex was observed in the unimodal model (*P*-value = .03) (**Table S3**). In the multimodal bagging model, males achieved a median TPD of 0.18 [0.04-0.66] versus females at 0.44 [0.09-0.86] (*P*-value = .02) (**Table S3**). This suggests the model’s prediction probabilities are higher (near to 1) for males than females. Race at both sites, showed no significant disparities (UoM *P*-value = .24; SU *P*-value = .51), despite varied medians. Ethnicity comparisons revealed no significant differences (UoM *P*-value = .58; SU *P*-value = .20) (**Table S3**). No additional biases were identified.

## Discussion

This multi-site study developed and validated ensemble learning approaches combined with resampling techniques, demonstrating that a bagging-based ensemble model using both under- and over-sampling accurately identifies patients with and without glaucoma. Incorporating unstructured clinical data with traditional structured data improves model’s ability to better distinguish patients with glaucoma from others without the disease, compared to models relying on only structured data elements alone. Training multiple learners with different sampling strategies addressed the class imbalance problem. Additionally, ensemble learning provided less biased predictions for sex, race and ethnicity sub-groups. Our approach can be used effectively to identify the presence or absence of various ocular and non-ocular phenotypes from EHR data.

Although, these multimodal models can help with more accurate cohort selection as compared to traditional models that use only ICD codes or only structured clinical data models,^1^ translation of such models is limited.^30^ To address this, we showed model explainability along with effectiveness and generalizability by performing an external site validation. Ensemble (bagging) of LR_ENN_ and LR_bSMOTE_ demonstrated stronger performance than stacking in both internal and external validation, as individually these models capitalize on their differing feature importance and SHAP values. The lower external validation performance as compared to the internal may be attributed to several factors. One key reason is the variations in clinical documentation practices, which is evident by varying clinical note lengths at the two sites. This suggests that relevant information may be recorded differently or may be documented in alternative locations, resulting in data inconsistencies. Furthermore, differences in patient characteristics, such as comorbidities, and disease severity, may also explain the observed drop in performance. Along with these variations, the external validation cohort may not fully represent the patient population seen at the internal site. These discrepancies highlight important challenges in applying predictive models to real-world settings. Moreover, the wide range of 95% confidence intervals underscores the need for larger annotated datasets in ophthalmology to improve model robustness and applicability.

Historical EHR healthcare data comes with inherent biases^31^^,^^32^ A study by Ravindranath et al showed that the inclusion of sensitive attributes (sex, race, and ethnicity) led to varied ML models fairness in predicting which glaucoma patients will progress to the point they will need surgical interventions, highlighting the need for fairness evaluations before model deployment.^19^ The developed ensemble models demonstrated consistent and less biased predictions across diverse subgroups of the patient population as compared to traditional models, except for patients at UoM for race and ethnicity. One potential reason for observed biases is that the UoM patient population is mainly non-Hispanic. Other glaucoma studies also have had limited numbers of Latinx patients.^33^ One approach to mitigate the model biases is understanding and removing potential proxy variables responsible for biases and model tuning,^34^^,^^35^ which would strengthen the argument for effectiveness of such models.

Depending upon clinical utility, threshold value needs to be set for the ML models.^35^ Additionally, the presence of several metrics which are based on the threshold probability makes it difficult to understand model biases. This study provides TPD, a different insight into bias assessment evaluation by proposing target probability difference, an approach to understand the confidence of model predictions and to address the limitations of single-threshold fairness metrics.^36^ The approach is useful in the healthcare context where the identification of target patients and having the confidence in the classification probabilities can be of utmost importance. TPD's threshold-independent evaluation revealed that racial and ethnic disparities in identifying patients with and without glaucoma were statistically insignificant, however, varied medians may indicate or suggest undetected biases either due to missing data or less representation of certain subgroups^37^^,^^38^ due to low glaucoma prevalence. By integrating TPD with standard metrics, we extend the evaluation of model fairness providing a deeper understanding of model fairness and prediction accuracy for equitable ML modeling and clinical utility.^39^

Glaucoma is one of the most common causes of irreversible blindness and a relatively low overall prevalence^15^ contributes to data imbalance when training of AI/ML models. Models with high false positives rates may inadvertently lead to unnecessary testing and unwarranted treatments. This necessitates the selection of the best off-the-shelf ML models,^40^ for specific disease-related clinical datasets. Most of the ML algorithms prioritize minimizing training errors, which may limit their ability to learn different aspects of the problem. Models like RF and SVC often struggle with imbalanced or noisy data, leading to overfitting or poor performance for minority cases,^25^^,^^41^^,^^42^ which was also evident in our study. Our findings suggest that targeted resampling techniques within ensemble frameworks can mitigate these issues, thereby leading to more accurate models with fair classifications. In this context, we found that bagging offers distinct advantages over stacking. While stacking can lead to overfitting due to its reliance on a meta-classifier trained on a potentially small subset of data, bagging enhances model robustness by aggregating predictions from multiple base learners, which helps to reduce variance and improve generalizability. Additionally, the advantage of using BioClinicalBERT, a specialized variant of BERT that has been fine-tuned on clinical text, is that it is adept at understanding medical language and terminology for clinical applications. Given the data from two different centers, the [CLS] token in BioClinicalBERT served as a pooled representation of the given input sequence saving efforts of further fine-tuning. Although further fine-tuning is possible which we feel is out of scope of this study.

Our study has several limitations. Although multiple notes were available for each patient, only the most recent note was used, based on the assumption that it would capture the most clinically relevant information, especially when combined with SDE. This approach also streamlined data processing by reducing redundancy and minimizing potential inconsistencies from outdated or conflicting documentation. The use of bSMOTE creates synthetic samples which may not align with the actual clinical context or representation of real-world data. Limited number of samples for internal validation dataset, and demographic heterogeneity within the patient population at the two sites constrained a comprehensive model fairness evaluation. One may consider using data-centric approaches to address class imbalance and mitigate biases before model training.^43^ To maintain model’s generalizability and avoid overfitting to one specific dataset, we did not modify the hyperparameters during model training which can be considered as future work. Moreover, only one specific threshold value was used to perform model validation.

In summary, we present a robust model capable of accurately identifying the patients with and without glaucoma in real-world data. Not only we are able achieve excellent precision and recall, but our model also demonstrated fairness across different demographic subgroups. Using approaches outlined here to deal with class imbalance will permit researchers to properly identify the patients with different ocular phenotypes. It will augment the use of real-world data to answer clinical questions and identify the patients with conditions of interest for recruitment into clinical trials and research studies.

## Supplementary Material

ooaf080_Supplementary_Data

## Data Availability

The datasets generated and analyzed during the study are not available due to privacy and ethics restrictions.
