# Supplementary material for: Ensemble learning to enhance accurate identification of patients with glaucoma using electronic health records
Source: JAMIA Open. 2025 Aug 10;8(4):ooaf080. doi: 10.1093/jamiaopen/ooaf080 (PMC12342940; doi:10.1093/jamiaopen/ooaf080)
Supplement: ooaf080_Supplementary_Data [file ooaf080_supplementary_data.zip › Supplementary_File.docx]

**Supplementary**

**Figure Legends:**

**Figure S1:** Labeling flowchart.

Note 1: Labels were generated by 2 board certified ophthalmologists by reviewing all the clinical data in the EHR. Third reviewer acted as a adjudicator where the results were discordant between first 2 reviewers.

Note 2: “Possible” glaucoma is capturing the cases that ophthalmologist could not decide if the patient did or did not have glaucoma based on the clinical data. This is different from “glaucoma suspect” who are patients with risk for glaucoma but do not have definitive clinical damage present.

**Figure S2:** Words per label for University of Michigan free text of the clinical note

**Figure S3:** Words per label for Stanford University free text of the clinical notes

**Figure S4:** Model classification capacity across patients with glaucoma and non-glaucoma. UoM: University of Michigan; SU: Stanford University; LR: Lasso Regression; ENN: EditedNearestNeighbours; bSMOTE: Borderline Synthetic-Minority Oversampling Technique. Note: The extension of the violin plot below 0 or above 1 does not represent negative or greater than 1 probability values but a feature of kernel density estimation used to indicate data distribution.

**Figure S5:** Density distribution of glaucoma across each category among all non-continuous features. UoM: University of Michigan; SU: Stanford University

**Tables**
