# Supplementary figures and images for: Ensemble learning to enhance accurate identification of patients with glaucoma using electronic health records
Source: JAMIA Open. 2025 Aug 10;8(4):ooaf080. doi: 10.1093/jamiaopen/ooaf080 (PMC12342940; doi:10.1093/jamiaopen/ooaf080)

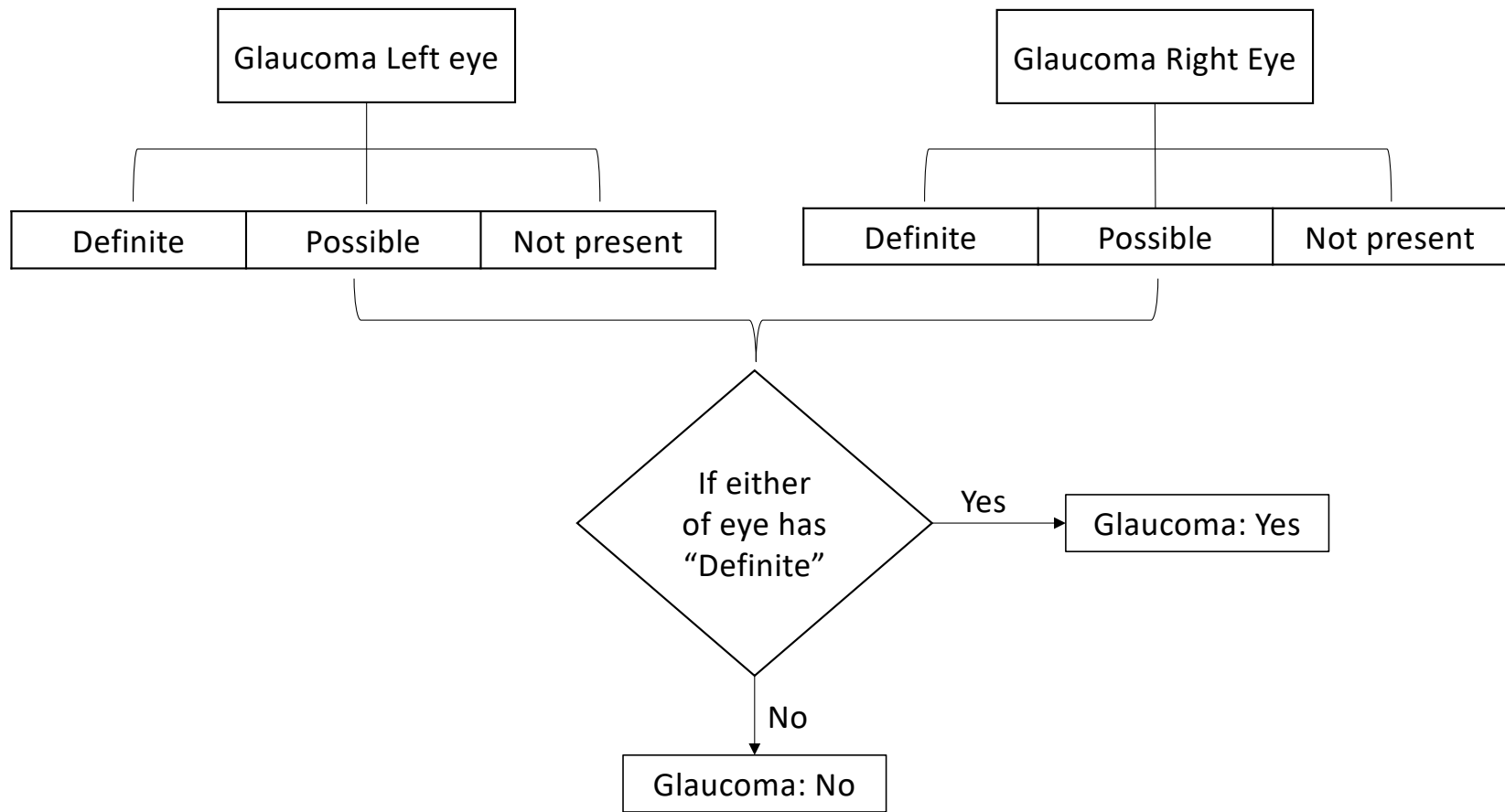

Supplement: ooaf080_Supplementary_Data [file ooaf080_supplementary_data.zip › Figure_S1.pdf]

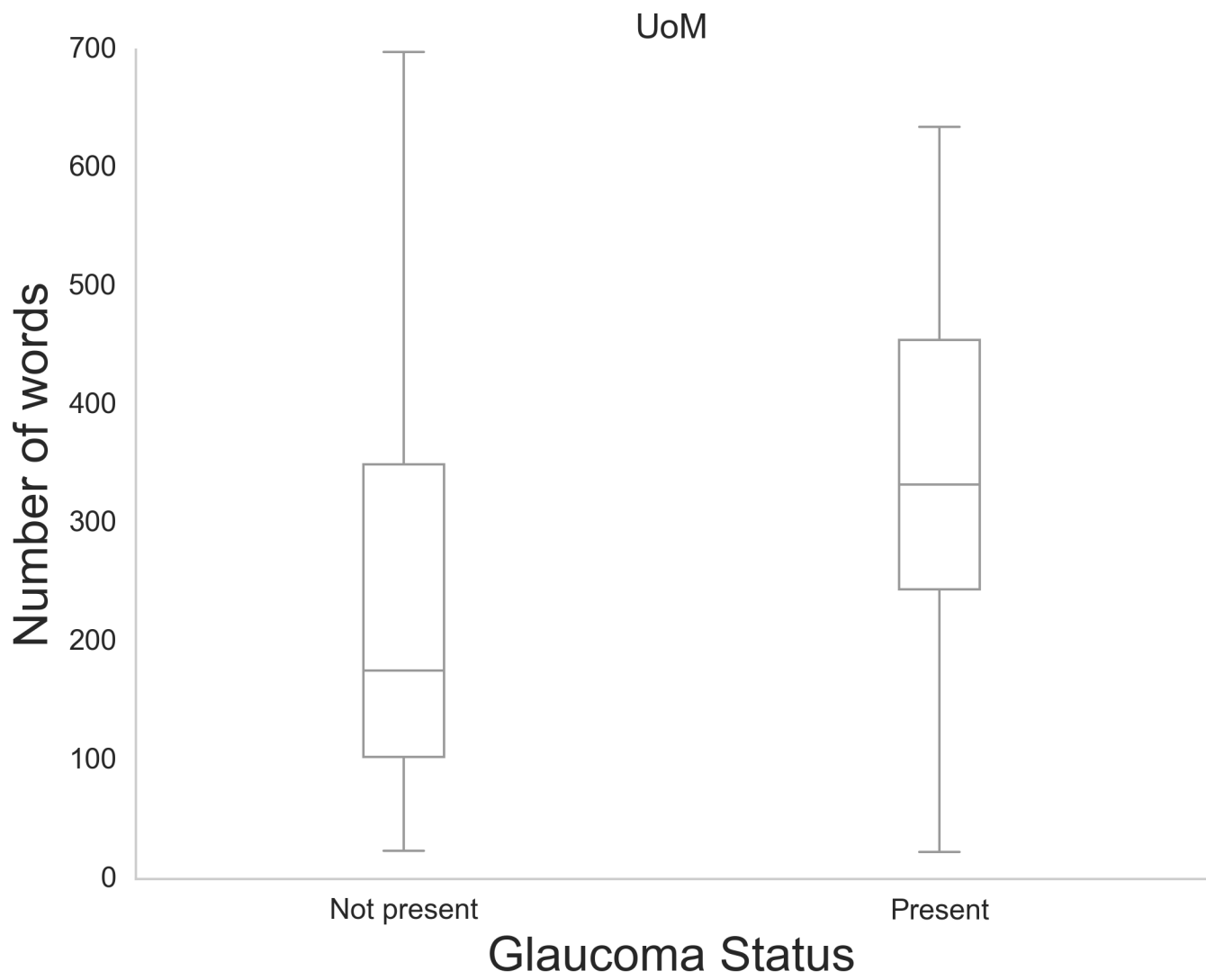

Supplement: ooaf080_Supplementary_Data [file ooaf080_supplementary_data.zip › Figure_S2.pdf]

SU

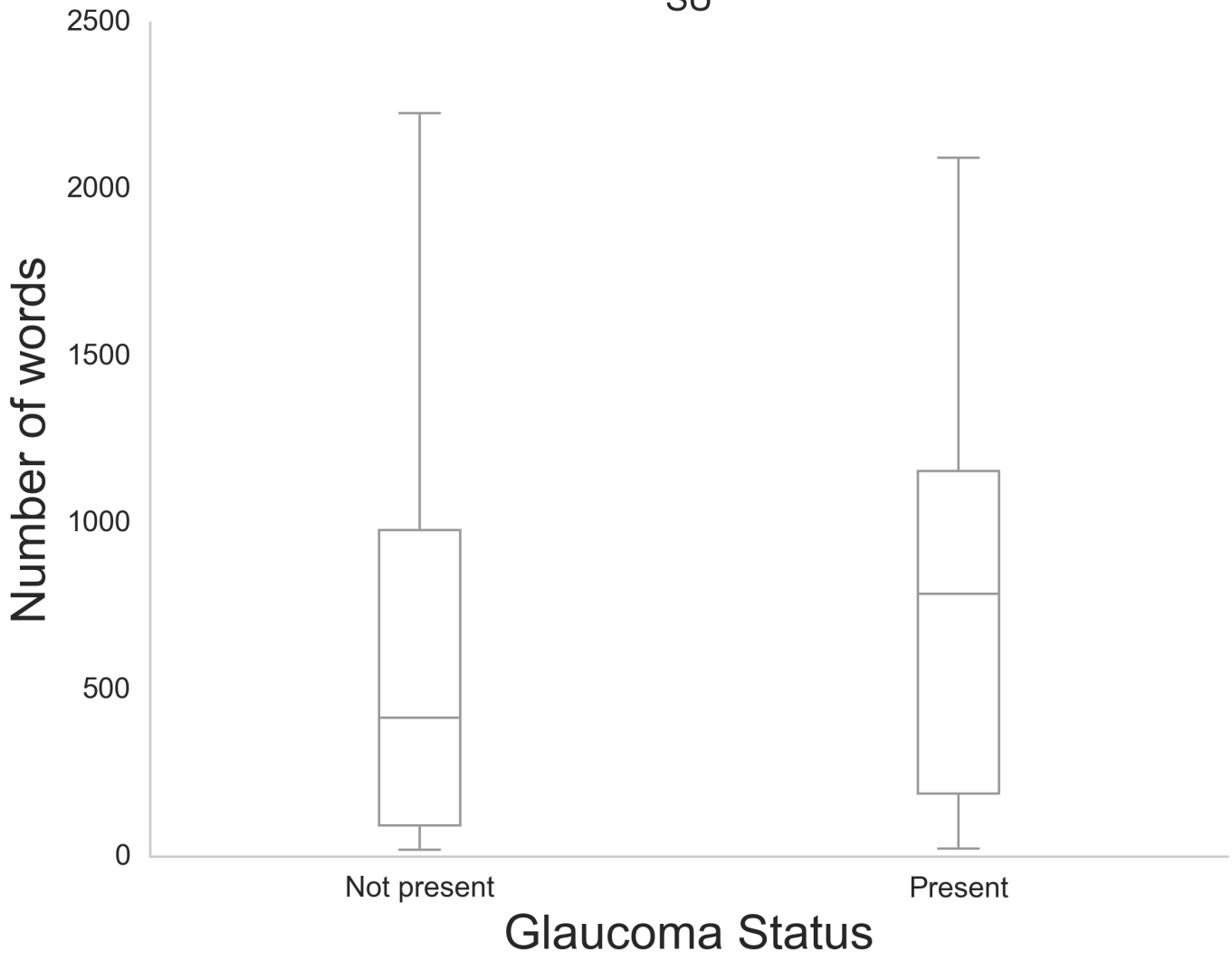

Supplement: ooaf080_Supplementary_Data [file ooaf080_supplementary_data.zip › Figure_S3.pdf]

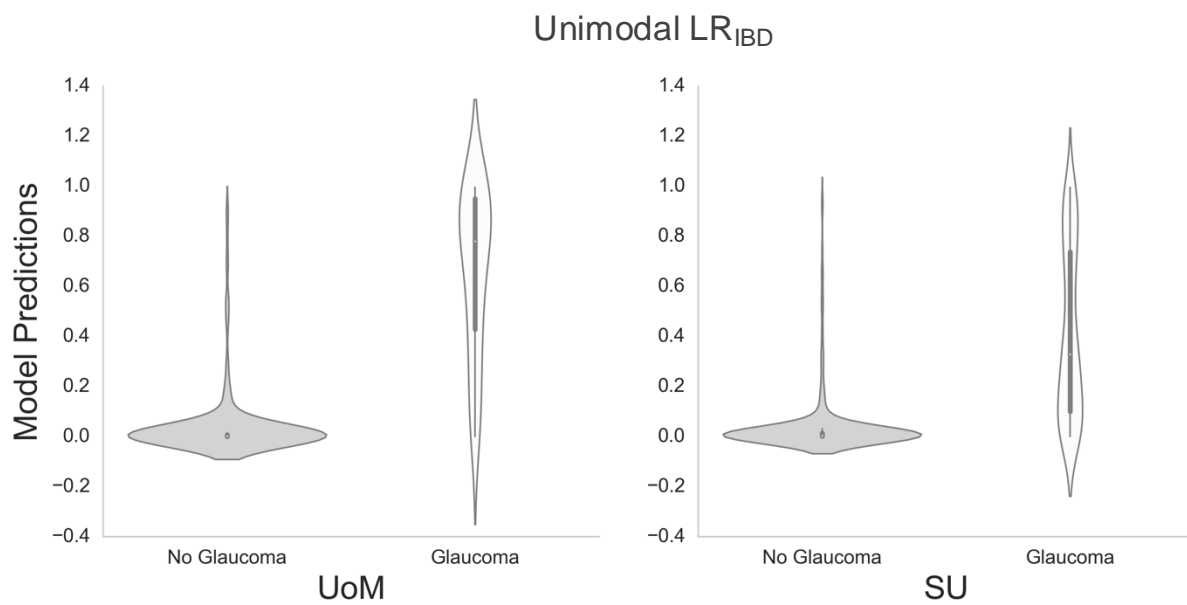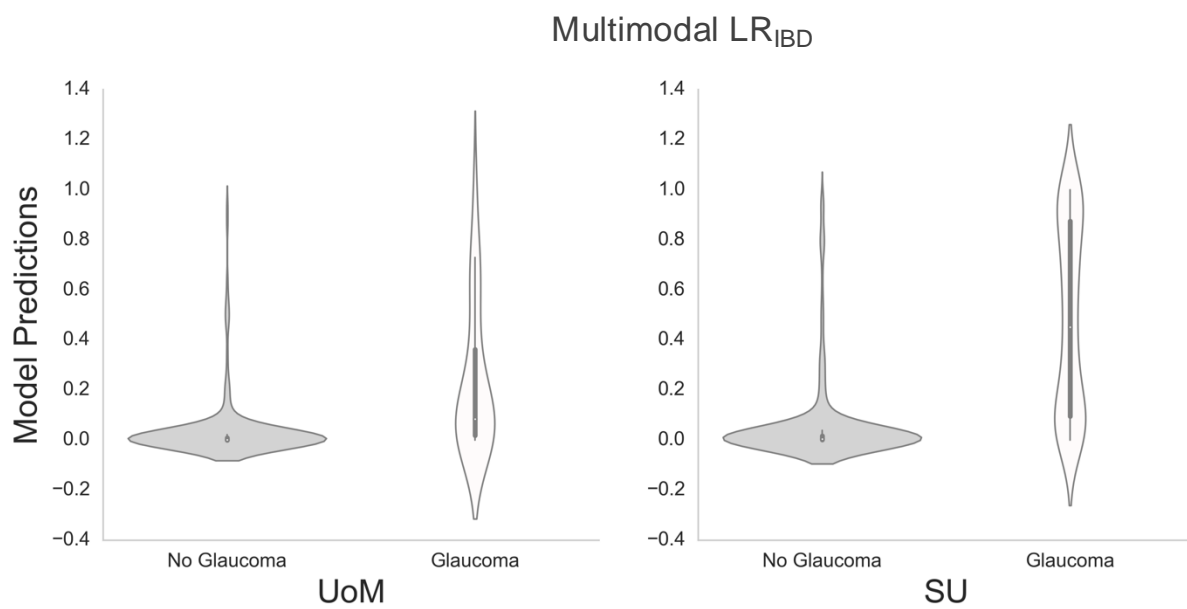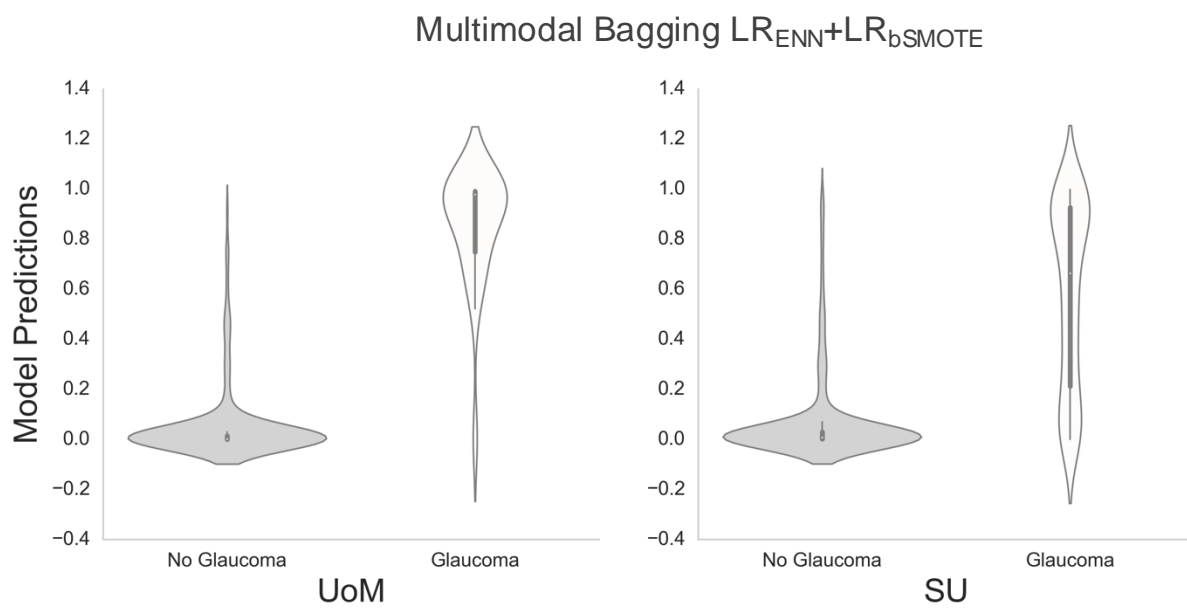

Supplement: ooaf080_Supplementary_Data [file ooaf080_supplementary_data.zip › Figure_S4.pdf]

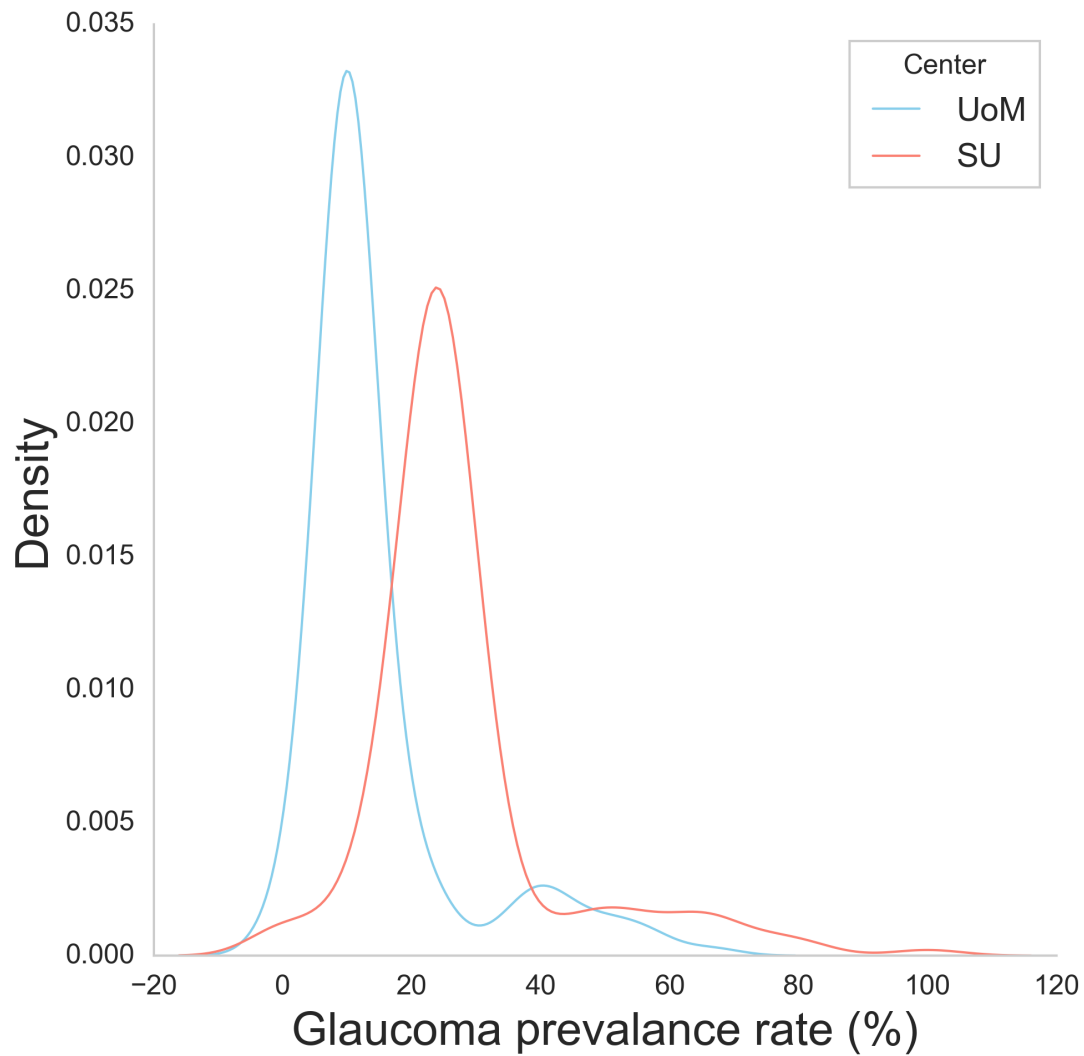

Supplement: ooaf080_Supplementary_Data [file ooaf080_supplementary_data.zip › Figure_S5.pdf]
